# Improving Web Searches: Case Study of Quit-Smoking Web Sites for Teenagers

**DOI:** 10.2196/jmir.5.4.e28

**Published:** 2003-11-14

**Authors:** Malcolm Koo, Harvey Skinner

**Affiliations:** ^1^Department of Public Health SciencesUniversity of TorontoToronto ONCanada

**Keywords:** Internet, smoking cessation, teens, teenagers, search engines, Web page analysis

## Abstract

**Background:**

The Web has become an important and influential source of health information. With the vast number of Web sites on the Internet, users often resort to popular search sites when searching for information. However, little is known about the characteristics of Web sites returned by simple Web searches for information about smoking cessation for teenagers.

**Objective:**

To determine the characteristics of Web sites retrieved by search engines about smoking cessation for teenagers and how information quality correlates with the search ranking.

**Methods:**

The top 30 sites returned by 4 popular search sites in response to the search terms "teen quit smoking" were examined. The information relevance and quality characteristics of these sites were evaluated by 2 raters. Objective site characteristics were obtained using a page-analysis Web site.

**Results:**

Only 14 of the 30 Web sites are of direct relevance to smoking cessation for teenagers. The readability of about two-thirds of the 14 sites is below an eighth-grade school level and they ranked significantly higher (Kendall rank correlation, tau = -0.39, *P*= .05) in search-site results than sites with readability above or equal to that grade level. Sites that ranked higher were significantly associated with the presence of e-mail address for contact (tau = -0.46, *P*= .01), annotated hyperlinks to external sites (tau = -0.39, *P*= .04), and the presence of meta description tag (tau = -0.48, *P*= .002). The median link density (number of external sites that have a link to that site) of the Web pages was 6 and the maximum was 735. A higher link density was significantly associated with a higher rank (tau = -0.58, *P*= .02).

**Conclusions:**

Using simple search terms on popular search sites to look for information on smoking cessation for teenagers resulted in less than half of the sites being of direct relevance. To improve search efficiency, users could supplement results obtained from simple Web searches with human-maintained Web directories and learn to refine their searches with more advanced search syntax.

## Introduction

The World Wide Web, with over 3 million public Web sites and over 1.4 billion Web pages [1], has become an important and influential source of health information [2]. In September 2002, there were an estimated 605 million people online worldwide [3]. In the United States, 90% (48 million) of the children and adolescents between the ages of 5 and 17 use computers, and 75% of the 14 to 17 year olds use the Internet [4]. With the vast amount and dynamic nature of information on the World Wide Web, it is not surprising to find that over 75% of those online use search sites to navigate the Web [5]. However, the amount of results returned from a search is often overwhelming. For example, 115000 results were found with the search terms "teen quit smoking" in Google.

Of the several thousand search sites or directories [6], only a few are of high popularity as indicated by their audience reach and time spent on them [7]. Although Google will provide up to a thousand results from a query, few users are likely to examine them all. In an observational study on 16 adult subjects, only 9 participants ever looked beyond the first search pages and only 5 of them ever clicked a link on those pages [8]. A survey done in 2002 on 1403 e-mail participants showed that only 23% of the users went beyond the second page [9]. Another pilot study of 12 teenagers found they looked past the fourth page of results less than 5% of the time [10]. Thus, position ranking in Web-search results, especially on the first few pages, is an important determinant of information accessibility by users.

Several studies have reported substantial variability in health-related Web-site content [11- 14]. While guidelines for evaluating the quality of health information on the Web are available [15- 19], the correlation between these guidelines and accuracy of health information is debated [20- 22]. Position ranking in search results was not associated with content quality [23]. Using the search term "breast cancer," Meric et al [24] reported that popularity of Web sites was associated with type rather than quality of content. In a sample of 75 Web sites that provided information on urinary incontinence, the Internet popularity indexes—as measured by the number of links to the main incontinence page of each Web site and by the number of links to all pages of each Web site divided by the number of pages of the site—were not correlated with a quality score based on Silberg et al [16] and the HONcode principles [25].

The aim of this study was: (a) to identify the characteristics of Web sites with information on smoking cessation for teenagers that ranked in the top 30 positions in a typical Web search on popular search sites and (b) to evaluate the association between those characteristics and the position ranking for sites that are of direct relevance to smoking cessation for teenagers. The findings are relevant for improving consumer access to health information.

## Methods

This study was carried out from May 2003 through June 2003. Web sites with information on smoking cessation for teenagers were identified with 4 popular search sites using a specific search term. The characteristics of the identified sites were collected with a Web-site characteristic checklist; 2 raters evaluated each Web site independently (details below).

### Search Protocol

Four popular search sites (Table 1) were used in this study. Users spend over 5 million search hours per month at each site. A search hour equals the number of visitors to a site multiplied by the average number of hours each visitor is estimated to have spent at the site.

**Table 1:** Popular search sites in the United States*

**Search Site**	**URL**	**Total Search Hours (Millions of Hours) in January 2003**	**MainUnderlying SearchEngine**
Google	www.google.com	18.7	Google
AOL	search.aol.com	15.5	Google
Yahoo!	www.yahoo.com	7.1	Google and Overture (for paid listings)
MSN	search.msn.com	5.4	LookSmart, Inktomi, Microsoft proprietary editor, and Overture (for paid listings)

^*^ Source: Search Engine Watch [26].

The search term on smoking cessation for teenagers was selected based on information from the Overture Search Term Suggestion Tool [27] and the 7search Keyword Suggestion Tool [28]. These sites provide a count of the search terms that were submitted to their search engines. Overture provides their search results to various popular search sites including Yahoo, MSN, AltaVista, Lycos, HotBot, and AllTheWeb [29]. For example, in April 2003 there were 40036 searches submitted to Overture with "quit smoking," 27812 with "stop smoking," and 9001 with "smoking cessation." Various other combinations of "teen," "youth," "adolescent," "quit smoking," "stop smoking," and "smoking cessation" were compared. Based on the frequency of searches performed on the Web as recorded by the Overture database, the search terms "teen quit smoking" were submitted to the 4 search sites to locate sites with information on smoking cessation for teenagers.

To mimic the search behavior of Web users, only the top 30 search results were included in the study. Sites ranking below the top 30 results are likely to be found only by more-persistent searchers [30]. Thirty results are equivalent to 3 pages (2 clicks) of the default number of results per page in Google and AOL, 2 such pages (1 click) in MSN, and one and a half such pages (one click) in Yahoo. The results from the 4 search sites were combined into one list to provide an overall picture of the search activity on the Web. The sites were reranked by first grouping the sites into 4 groups by the number of search sites that included them (1 to 4 search sites) and then by the position ranking provided by the search results within each group. The top 30 reranked sites formed the sample for the analysis.

Since the rankings of Web sites within search-site results change frequently, the search results were captured in spreadsheet format using the Google API Search Tool [31]. The Web pages of sites identified by search results were captured using Offline Explorer software [32] to facilitate the recall of the exact page content when necessary and to provide consistency for the 2 raters.

**Table 2:** Site characteristics and correlation with search ranking for 14 sites relevant to teenagers who are seeking information on smoking cessation

**Site Characteristic**	**Inter-Rater Reliability (Kappa)**	**Characteristics**	**Number (%)**	**Kendall Rank Correlation, Tau (*P* value)**
**Essential**				
Search feature in the site	0.86	Present	8 (57)	0.15 (.52)
		Absent	6 (43)	
Site navigation system on page	0.76	Present	11 (79)	-0.16 (.42)
		Absent	3 (21)	
Privacy statement	0.57	Present	8 (57)	0.18 (.41)
		Absent	6 (43)	
Disclaimer	0.69	Present	9 (64)	0.11 (.70)
		Absent	5 (36)	
Readability grade level	NA*	< 8.0	5 (36)	-0.39 (.05)
		≥ 8.0	9 (64)	
Broken links on page	NA	Present	7 (50)	0.08 (.75)
		Absent	7 (50)	
**Enhancement**				
Indication of sponsorship	0.19	Present	11 (79)	0.09 (.66)
		Absent	3 (21)	
Pop-up advertisements or banner advertisements	0.59	Present	4 (29)	0.18 (.17)
		Absent	10 (71)	
Contact e-mail address	0.51	Present	10 (71)	-0.46 (.01)
		Absent	4 (29)	
Phone number or mailing address	1.00	Present	4 (29)	0 (1.00)
		Absent	10 (71)	
Content on cessation method: behavioral approach	0.43	Present	11 (79)	0.31 (.10)
		Absent	3 (21)	
Content on cessation method: medication approach	0.84	Present	10 (71)	-0.43 (.02)
		Absent	4 (29)	
Content on cessation method: alternative approach	0.51	Present	5 (36)	-0.42 (.02)
		Absent	9 (64)	
Annotated external hyperlinks	0.72	Present	5 (36)	-0.39 (.04)
		Absent	9 (64)	
Interactive component (quiz, game, or bulletin board)	0.53	Present	8 (57)	-0.18 (.44)
		Absent	6 (43)	
Material in video or audio format	1.00	Present	1 (7)	-0.20 (.31)
		Absent	13 (93)	
**Technical**				
Page size (kilobyte)	NA	< 35	6 (43)	-0.39 (.04)
		≥ 35	8 (57)	
Meta description tag	NA	Present	8 (57)	-0.48 (.002)
		Absent	6 (43)	
Meta keywords tag	NA	Present	11 (79)	-0.31 (.13)
		Absent	3 (21)	
Persistent cookies	NA	Present	3 (21)	-0.34 (.06)
		Absent	11 (79)	
Part of a larger Web site	0.72	Yes	6 (43)	0.03 (.90)
		No	8 (57)	
Link density (reverse links)	NA	1	6 (43)	-0.58 (.02)
		2-100	4 (29)	
		> 100	4 (29)	

^*^ NA = Not applicable. Kappa values for these characteristics were not available because they were analyzed by the WebXact Watchfire Page Analysis [35], except for readability grade level which was evaluated by only 1 rater.

### Checklist of Web-Site Characteristics

A checklist was uses to evaluate the characteristics of the Web sites (see Table 2 for checklist items). The readability was estimated by the Flesch-Kincaid grade-level score [33]. (The Flesch-Kincaid grade-level score rates text on a United States grade-school level. For example, a score of 8.0 means that an eighth grader can understand the document.) Sample passages from the Web pages with information pertaining to smoking cessation of the identified sites were pasted into Microsoft Word XP for Windows to obtain the score. The results were recorded in a spreadsheet and subsequently imported into SPSS [34] for analysis. The number of broken links, page size, presence of meta tags, and presence of persistent cookies were obtained from WebXact Watchfire Page Analysis [35]. (Meta tags are HTML [hypertext markup language] tags that provide information about the content of a Web page for indexing by search engines but do not affect how a Web page is displayed by a browser.) Link density was obtained by using a reverse-lookup query (link:siteURL, where *siteURL* is replaced by the Web site's URL) in Google. The link density of a site is the number of external sites that have a link to that site. A site with a higher link density is generally more likely to be found by visitors because they may find it through the external sites.

### Statistical Analysis

Correlations between position ranking and the Web-site characteristics were calculated using the Kendall rank correlation. The value of the coefficient (tau) ranges from -1 to 1. A value of zero indicates no correlation, values near 1 indicate a strong direct correlation, and values near -1 indicate a strong inverse correlation. Interobserver reliability between the 2 raters was calculated using Kappa statistics on all variables except readability, link density, and those returned by WebXact Watchfire Page Analysis. We regarded *P*£ .05 as statistically significant.

## Results

Of the top 30 sites identified by the 4 search sites using the search terms "teen quit smoking," only 14 were relevant to teenagers who are seeking information on smoking cessation. We also evaluated the search results from Google by using other similar search terms. The number of relevant sites ranged from 5 to 17 (Table 3). Although we used only 1 search site to illustrate the effect of search terms on the type of Web sites found, the result should be similar at other search sites.

### Characteristics of the 14 Relevant Web Sites

The characteristics of the 14 sites are summarized in 3 categories (Table 2).

#### Essential-Characteristic Category

The essential-characteristic category contains those characteristics that contribute to user dissatisfaction if absent or inadequately provided. The presence of a privacy statement and disclaimer, although it appears not to be required for the functioning of a Web site, wasreported to be essential in a Web-user interface study [36].

The correlation between the 2 raters ranged from 1.00 for 2 characteristics (presence of phone number or mailing address and presence of material in video or audio format) to 0.19 for indication of sponsorship. The median correlation was 0.69 for the 15 characteristics evaluated by both raters.

In the essential category, 8 sites (57%) contained a site-search feature and 11 sites (79%) contained links for navigation in the site. However, 2 sites contained neither of the features. Over half of the sites contained either a privacy statement (57%) or a disclaimer (64%) but only a third of the sites contained both. About one-third of the sites have readability below eighth-grade school level and they ranked significantly higher (tau = -0.39, *P*= .05) than those that have readability above or equal to that level. The median grade level was 8.5. Half the sites contained one or more broken internal or external hyperlinks.

#### Enhancement-Characteristic Category

In the enhancement-characteristic category, 11 sites (79%) indicated their sponsorship. Apparently because most of the sites were sponsored by organizations, government bodies, or educational institutions, only 4 sites (29%) had either pop-up advertisements or in-page banner advertisements. E-mail address (71%) was the most-common contact information available while phone number or mailing address was present in 29% of the sites. Sites that ranked higher were significantly associated with the presence of e-mail address for contact (tau = -0.46, *P*= .01). Eleven sites (79%) had information on behavioral approach as a method of smoking cessation. Ten sites (71%) had information on a medication (nicotine replacement) approach, and 5 sites (36%) had information on alternative approaches such as acupuncture, hypnosis, laser therapy, and herbal cigarettes. Both the presence of medication (tau = -0.43, *P*= .02) and alternative approaches (tau = -0.42, *P*= .02) were significantly associated with a higher search ranking. Five sites provided annotated hyperlinks to external sites and their presence was significantly associated with a higher search ranking (tau = -0.39, *P*= .04). Eight sites contained interactive components such as quizzes, games, or bulletin boards. Only 1 site provided material in video or audio format.

**Table 3:** Type of Web sites found with different search terms using Google search site

**Type of Web Site**	**Search Terms Used**				
	teen quit smoking	teen stop smoking	teen smoking cessation	youth quit smoking	adolescent quit smoking
Site with information to help teenagers quit smoking	14	5	5	17	5
Page with hyperlinks to Web sites with information to help teenagers quit smoking	3	1	5	3	4
News or press release	4	5	3	3	5
Report of study results or proceedings from conferences	1	2	5	2	9
Recruitment of study subjects	2	1	1	0	0
Commercial site	3 (2 were redirects*)	4 (1 was a redirect)	0	0	2 (both redirects)
Site for teenagers but not on smoking	1	2	0	0	0
Resources on teenager smoking cessation for parents or health professionals	1	4	5	3	2
Health organizations or community centers	0	1	3	1	1
Page not found	1	4	2	1	0
Other	0	1 (alt.support.stop-smoking Usenet archive)	1 (porno-graphic Web site)	0	2 (mental health Web site)

^*^ The visitor was automatically sent to a page other than the page listed in the search results (see Discussion for details).

#### Technical-Characteristic Category

In the technical-characteristic category, the largest file size of the landing page (the page reached when clicking on the search-site result) was 134 kilobytes, which is equivalent to approximately 19 seconds of download time on a 56 Kbps modem. Sites that were equal to or larger than 35 kilobytes (57%) were ranked significantly higher (tau = -0.39, *P*= .04) by the search sites. Eight (57%) and 11 (79%) of the sites had meta description and meta keywords tags, respectively. The presence of a meta description tag was significantly associated with a higher search rank (tau = -0.48, *P*= .002). Although 5 sites used cookies (small files sent to the browser along with a Web page for tracking a visit), only 3 of them used a persistent cookie that is stored on the user's hard disk and 4 used a session cookie that is automatically deleted from the browser's cache when the browseris closed. Six (43%) sites were just part of larger Web sites containing information other than smoking. The median link density of the 14 Web pages was 6 and the maximum was 735. A higher link density was significantly associated with a higher search rank (tau = -0.58, *P*= .02).

## Discussion

The key finding of this study was that using simple search terms on popular search sites to look for information on smoking cessation for teenagers, less than half (14 of 30) of the sites found were of direct relevance. The remaining sites were study reports, news, and hyperlinks.

We did not include all information retrieved from Web searches, as has been done in studies on other topics [37], since users tend not to go beyond the first few pages of search results [9,10]. Instead, we evaluated only the top 30 search results to mimic typical Web search behavior.

Searching with the terms "teen quit smoking" on 7 popular search sites, Edwards et al [38] also reported that only 40% of the 140 potential hits were focused on cessation. In our study, 1 site of pornographic nature was found when using the search terms "teen smoking cessation" but no such sites were found when using the search terms "teen quit smoking" in contrast to a previous report [39] where 7 out of the top 20 sites were teen pornography sites.

Of public health concern was the finding that 3 sites were commercial sites and 2 of them were linked back to a single online drug store using a page-redirect spamming technique. With page redirection, an optimized page with unique and specific terms is submitted to search sites with the single purpose of ranking high on a specific topic. However, anyone clicking the link to this page is automatically sent to a real destination page, which often contains material unrelated to the initial search terms. For example, one site used "what-happens-to-your-body-when-you-quit-smoking.htm" as the name of its Web page. However, this page contains no information on smoking cessation. Instead, it is a page with a JavaScript that immediately redirects visitors to an online drug store.

Several important associations were found between Web-site characteristics and position ranking in the top 30 search results. These results can be used for optimizing site development in future smoking-cessation Web sites.

### Essential-Characteristic Category

As an example of how these results can be used, of the 6 items in the essential-characteristic category, readability (lower grade level) was associated with higher position ranking. The lack of search box, navigational menu, privacy statement, or disclaimer, or the presence of broken links, was not uncommon, but their absence was not associated with lower position ranking.

### Enhancement-Characteristic Category

In the enhancement-characteristic category, presence of contact e-mail address, medication-cessation information, alternative-approach information, and annotated external links were associated with higher position ranking. It is surprising to find that only 1 site displayed a HONcode insignia which, along with the associated membership, is an indication that a site complies with an 8-point code of conduct put forth by Health on the Net [18]. Although 73% of young people said that knowing who produced health information is very important to them, only 29% of those who looked up health information online checked the source the last time they conducted a search [5] and it is likely that fewer will check for the authenticity (for example, verify the membership status of a site at the HON Web site) of any indications of external recognition even if they are present [8].

### Technical-Characteristic Category

In the technical-characteristic category, page size that was larger than 35 kilobytes, presence of a meta description tag, and a high link density were associated with higher ranking. The strong association between site description meta tag and ranking (tau = -0.48, *P*= .002) suggests that such information is relevant to the ranking algorithms of the search-engines used. Including a concise description tag is likely to be more effective in improving search-engine visibility than just a comprehensive keywords list. In fact, due to high rate of keyword repetition and spam, search sites such as Google and AltaVista do not give consideration to the keywords meta tag in their ranking [40,41]. As expected, link density is strongly associated with ranking (tau = -0.58, *P*= .02). Search engines generally use the number of incoming links (link density) in their ranking algorithm. However, Google's PageRank algorithm also takes into account the number of outgoing links on the page of each of the incoming links [42].Therefore, to achieve a high ranking a Web site should try to get listed on as many sites as possible and, in particular, on those sites that have as few external links as possible. Since search engines assign higher ranking to sites with incoming links that originate from pages containing fewer external links, and sites with annotated external links tend to have fewer links than those sites without annotated external links, this may explain the association between the presence of annotated external links and higher ranking (tau = -0.39, *P*= .04).

To improve search efficiency, users may want to supplement results from search sites with those from subject-based Web directories that are created and maintained by people, rather than by algorithms, such as Yahoo! Directory, which has a teen-smoking section [43]. Using the Yahoo! directory, we found 25 sites listed, of which only 4 were found using our search terms at the 4 popular search sites. In addition, users may want to learn and apply the specific syntax of their favorite search sites when searching for information. For example, quit-smoking Web sites of the commercial (.com) domain can be eliminated from the search results by entering "quit smoking -site:.com" in the search box in Google.
